# A prediction score model of difficult endoscopic submucosal dissection for early esophageal cancer and precancerous lesions

**DOI:** 10.1080/07853890.2024.2446706

**Published:** 2024-12-30

**Authors:** Li Gao, Jing Su, Hailong Ding, Haisheng Qian, Guoxin Zhang, Jin Yan

**Affiliations:** a Department of Gastroenterology, The First Affiliated Hospital with Nanjing Medical University, Nanjing, China; b The First Clinical Medical College, Nanjing Medical University, Nanjing, China; c Department of Gastroenterology, Xuzhou Central Hospital, Xuzhou, China; d Department of Information, The First Affiliated Hospital with Nanjing Medical University, Nanjing, China

**Keywords:** Endoscopic submucosal dissection, clinical score model, technical difficulty, early esophageal cancer, precancerous lesion

## Abstract

**Background:**

To establish a prediction model to triage difficult esophageal endoscopic submucosal dissection (ESD) in order to reduce complications.

**Methods:**

This retrospective study enrolled 742 patients who underwent ESD for early esophageal cancer and precancerous lesions. Difficult ESDs were defined as those meeting any of the following criteria: (1) long procedure time (≥ 90 min), (2) occurrence of intraoperative adverse events (muscularis propria injury, perforation, and massive hemorrhage), or (3) piecemeal resection. Logistic regression analysis was performed to identify the predictors of technical difficulty. Then we developed a score prediction model according to the Framingham system for the first time and presented a convenient table containing the risk probability for each patient. In addition, the score model was validated in the validation cohort.

**Results:**

The risk factors for difficult ESDs were as follows: lesion total length 4–6 cm (1 point), 7–9 cm (2 points) or ≥10 cm (3 points); lesion ≥ 2/3 circumference (2 points); preoperative invasion depth extending to submucosa (2 points). Areas under ROC curves for the score model in the derivation and validation sets were 0.768 (95% CI, 0.726–0.811) and 0.719 (95% CI, 0.646–0.793), respectively. In addition, the percentage of difficult ESDs in easy (score = 0), intermediate (score = 1–2), difficult (score = 3–4), and very difficult (score ≥5) categories were 14.8%, 36.1%, 62.6%, and 87.5% in the derivation cohort and 14.8%, 33.7%, 56.3%, and 68.8% in the internal validation cohort.

**Conclusions:**

We successfully established a prediction model to assess the difficulty of esophageal ESD and it can provide a scientific basis for clinical management to reduce complications.

## Introduction

Esophageal cancer has a very poor prognosis among the gastrointestinal tumors because of difficulties in the early detection [1]. Recently, with the continuous improvement of endoscopic diagnostic techniques, the diagnosis rate of early esophageal cancer has been greatly enhanced. Endoscopic submucosal dissection (ESD) has emerged as a promising technique for the treatment of early esophageal cancer and precancerous lesions. Compared with conventional endoscopic mucosal resection (EMR), ESD provides en bloc specimens for adequate pathological evaluation, thus achieving a higher curative resection rate and a lower rate of local recurrence [2]. However, esophageal ESD is technically difficult with a longer procedure time and a higher incidence of complications than EMR [3]. If the difficulty of ESD could be predicted preoperatively, this would assist us in rationalizing surgical planning, preventing complications, and optimizing training programs for ESD technicians.

Several reported prediction models have been used to assist in forecasting the technical difficulty of ESDs for gastrointestinal tumors preoperatively. A previous study developed a risk scoring system of difficult ESD for remnant gastric cancer after distal gastrectomy [4]. Another research constructed a predictive nomogram of difficult ESD for colorectal neoplasms [5]. Due to the narrow lumen and thin wall of the esophagus, which is susceptible to respiratory and cardiac pulsations, esophageal ESD is more difficult and more likely to require preoperative difficulty prediction. However, there have been no validated tools available to predict technically difficult ESD for esophageal cancer and precancerous lesions. Therefore, our study sought to establish a clinical scoring model to stratify the difficulty of ESD for early esophageal cancer and precancerous lesions.

## Materials and methods

### Data collection

Patients with early esophageal cancer or intraepithelial neoplasia who underwent ESD treatment at Jiangsu Province Hospital from January 2019 to June 2022 were evaluated from the original endoscopic database system. Inclusion criteria for this study were as follows: (1) pathological diagnosis of early esophageal cancer or intraepithelial neoplasia; (2) CT showed no lymph node metastasis; and (3) patients were treated with ESD and the lesions were successfully removed from the esophagus. We excluded patients who received treatment for other lesions besides the esophagus during surgery which will interfere with the exact time of the ESD procedure. In total, 742 patients who met the criteria were enrolled in this study.

To establish a predictive scoring model for the difficulty of esophageal ESD, we performed a retrospective analysis of registry data, including demographics (age, sex, basic diseases, personal history, and family history), endoscopic findings (length, number, location, circumference), procedure time, intraoperative adverse events (AEs) and depth of infiltration obtained from preoperative endoscopic ultrasonography (EUS). The study was conducted in accordance with the Declaration of Helsinki and has been approved by the Ethics Committee of the First Affiliated Hospital of Nanjing Medical University (2022-SR-468). Written informed consent for ESD and privacy was obtained from all of the patients. We have fully anonymized them and ensured that this paper does not contain any data which can identify individual.

### ESD procedure

The day before the operation, each patient was scheduled for a CT scan and an EUS for preoperative ESD evaluation. All esophageal ESDs were performed by 7 strictly trained endoscopists to avoid man-made interference. Each endoscopists has five or more years of experience and has performed more than 120 ESDs. General anesthesia and mechanical ventilation have been adopted to reduce the effects of respiratory movements during surgery. The typical sequence of ESD procedures was as follows. Before ESD, an endoscopy was performed to estimate the tumor location and size. After the boundary between the lesion and normal mucosa was determined using 1% Lugol solution, a mixture of saline solution, adrenaline, and indigo carmine was injected into the tumor base to sufficiently elevate the target mucosa. An incision was made at the periphery of the tumor where the submucosal dissection began until complete removal. After ESD, preventive endoscopic hemostasis and clipping were performed for any oozing, exposed vessels, or injured muscularis propria.

### Definition

En bloc resection was defined as resection in one piece and intact retrieval from the oral cavity. ESD procedure time referred to the total time from marking dots around the tumor to the complete resection and management of intraoperative AEs. Difficult ESDs were defined as those meeting any of the following criteria: (1) long procedure time (≥ 90 min), (2) occurrence of intraoperative AEs (muscularis propria injury, perforation, and massive hemorrhage), or (3) piecemeal resection. The esophagus is divided into the following segments by the distance to the incisors: cervical segment (from the esophageal entrance to 18 cm); upper thoracic segment (18–24 cm); middle thoracic segment (24–32 cm); lower thoracic segment (32–40 cm); and ventral segment (from 40 cm to the esophagogastric junction). For esophageal lesions spanning two or more segments, we categorized them according to the location of their midpoints, especially when the midpoints were 18, 24, 32, and 40 cm, they were classified as the more superior segments. The depth of tumor infiltration was categorized into two groups according to whether the tumor invaded the submucosa or not.

### Model development and scoring algorithm

Enrolled patients were randomly divided into the derivation and validation sets in a 7:3 ratio. The relationships between risk factors and ESD difficulty were tested using logistic regression models in the derivation set. Candidate predictors with *p* < 0.05 in univariate analysis were included in logistic regression with forward stepwise selection. At the same time, we conducted collinearity diagnostics on the included predictors.

After the logistic regression model was finally determined, points were assigned to each predictor based on the weight coefficient and variable type according to the Framingham Study risk score functions. The specific steps were as follows: (1) organizing the risk factors into meaningful categories and determining reference values; (2) selecting the least risky state of each factor as the base category and allocating it a value of 0; (3) computing how far each category is from the base category in terms of regression units; (4) determining a constant value corresponding to 1 point in the scoring tool; (5) dividing the distance of each risk factor state by the constant and rounding the result to the nearest whole number to simplify scoring [6, 7]. The difficulty score for an individual ESD procedure was determined by the total point of each risk factor. Model performance was assessed by calculating the area under the ROC curve with the 95% confidence interval (CI). Then, the model was validated in the validation set.

### Statistical analysis

Means ± standard deviations, medians and interquartile ranges (IQR), or frequencies and percentages were used to describe the variables. Differences between groups were analyzed using the Student t-tests, the Wilcoxon rank-sum tests, chi-square tests, or Fisher exact tests, as appropriate. Statistical significance was indicated by *p* < 0.05. We then performed a logistic regression analysis of variables that were statistically significant in the univariate analysis. Risk factors with *p* < 0.05 were considered statistically significant and used to develop a clinical prediction model. A receiver operating characteristic (ROC) curve was constructed to evaluate the performance of the model. Data management and analysis were performed by IBM SPSS Statistics 25.

## Results

### Characteristics of the patients and tumors

For a total of 742 eligible patients, 524 patients were enrolled in the derivation set and 218 patients were included in the validation set. Table 1 illustrated the clinical and tumor characteristics of the enrolled patients. No significant differences were seen in demographic, endoscopic tumor findings, procedure time, intraoperative AEs, or depth of infiltration between the derivation and validation cohorts (Table 1).

**Table 1.** Baseline characteristics of the patients and tumors.

Variables	All (*n* = 742)	Derivation set (*n* = 524)	Validation set (*n* = 218)	*p* Value
Age, y	64.58 ± 7.80	64.62 ± 7.76	64.49 ± 7.91	0.837
Gender, n				0.194
Male	523(70.5)	362(69.1)	161(73.9)	
Female	219(29.5)	162(30.9)	57(26.1)	
Hypertension, n				0.811
Yes	243(32.7)	173(33.0)	70(32.1)	
No	499(67.3)	351(67.0)	148(67.9)	
Diabetes, n				0.250
Yes	61(8.2)	47(9.0)	14(6.4)	
No	681(91.8)	477(91.0)	204(93.6)	
Smoking history, n				0.387
Yes	265(35.7)	182(34.7)	83(38.1)	
No	477(64.3)	342(65.3)	135(61.9)	
Drinking history, n				0.941
Yes	230(31.0)	162(30.9)	68(31.2)	
No	512(69.0)	362(69.1)	150(68.8)	
Previous chemoradiotherapy, n				0.858
Yes	11(1.5)	7(1.3)	4(1.8)	
No	731(98.5)	517(98.7)	214(98.2)	
Family history, n				0.528
Yes	64(8.6)	43(8.2)	21(9.6)	
No	678(91.4)	481(91.8)	197(90.4)	
Lesion number, n				0.542
Single	659(88.8)	463(88.4)	196(89.9)	
Multiple	83(11.2)	61(11.6)	22(10.1)	
Total length of lesions, median (IQR)(cm)	4(3–6)	4(2–7)	4(3–6)	0.487
Cervical segment, n				1.000
Yes	2(0.3)	2(0.4)	0(0.5)	
No	740(99.7)	522(99.6)	218(99.5)	
Upper thoracic segment, n				0.572
Yes	68(9.2)	46(8.8)	22(10.1)	
No	674(90.8)	478(91.2)	196(89.9)	
Middle thoracic segment, n				0.180
Yes	443(59.7)	321(61.3)	122(56.0)	
No	299(40.3)	203(38.7)	96(44.0)	
Lower thoracic segment, n				0.421
Yes	283(38.1)	195(37.2)	88(40.4)	
No	459(61.9)	329(62.8)	130(59.6)	
Ventral segment, n				0.329
Yes	4(0.5)	5(1.0)	0(0)	
No	738(99.5)	519(99.0)	218(100)	
Circumference, n				0.360
<2/3	608(81.9)	425(81.1)	183(83.9)	
≥2/3	134(18.1)	99(18.9)	35(16.1)	
Preoperative invasion depth, n				0.403
m1–m3	645(86.9)	452(86.3)	193(88.5)	
sm	97(13.1)	72(13.7)	25(11.5)	
Intraoperative AEs, n				0.112
Muscularis propria injury, n	66(8.9)	41(7.8)	25(11.5)	
Perforation	0(0)	0(0)	0(0)	–
Massive hemorrhage, n	0(0)	0(0)	0(0)	–
En bloc resection, n	742(100)	524(100)	218(100)	–
Procedure time, n	60(45,90)	60(45,90)	60(45,90)	0.077
Depth of pathological infiltration, n				0.301
m1–m3	690(93.0)	484(92.4)	206(94.5)	
sm	52(7.0)	40(7.6)	12(5.5)	

### Risk factors for ESD difficulty

All patients achieved en bloc resections and none of them experienced serious adverse events such as ­massive hemorrhage, perforation, and pneumothorax. Muscularis propria injury was present in a total of 66 patients and was all successfully clipped. The 75^th^ percentile of the procedure time was calculated to be 90 min after the statistical description. We classified ESDs into a simple group (*n* = 327, 62.4%) and a difficult group (*n* = 197, 37.6%) according to the definition. Then, the risk factors for ESD difficulty were analyzed between two groups. Univariate analysis revealed that the risk predictors of difficult ESDs in the derivation set were lesion number, circumference, total length, and preoperative invasion depth (Table 2).

**Table 2.** Univariate analysis of risk factors for ESD difficulty in the derivation set.

Variables	Simple ESD *n* = 327	Difficult ESD *n* = 197	*p* Value
Age, y	64.48 ± 7.98	64.86 ± 7.39	0.587
Gender, n			0.710
Male	224 (68.5)	138(70.1)	
Female	103(31.5)	59(29.9)	
Hypertension, n			0.994
Yes	108(33.0)	65(33.0)	
No	219(67.0)	132(67.0)	
Diabetes, n			0.074
Yes	35(10.7)	12(6.1)	
No	292(89.3)	185(93.9)	
Smoking history, n			0.765
Yes	112(34.3)	70(35.5)	
No	215(65.7)	127(64.5)	
Drinking history, n			0.860
Yes	102(31.2)	60(30.5)	
No	225(68.8)	137(69.5)	
Previous chemoradiotherapy, n			0.918
Yes	5(1.5)	2(1.0)	
No	322(98.5)	195(99.0)	
Family history, n			0.784
Yes	26(8.0)	17(8.6)	
No	301(92.0)	180(91.4)	
Lesion number, n			0.002*
Single	300(91.7)	163(82.7)	
Multiple	27(8.3)	34(17.3)	
Total length of lesions, median (IQR)(cm)	3(2–5)	6(4–8)	<0.001*
Cervical segment, n			1.000
Yes	1(0.3)	1(0.5)	
No	326(99.7)	196(99.5)	
Upper thoracic segment, n			0.388
Yes	26(8.0)	20(10.2)	
No	301(92.0)	177(89.8)	
Middle thoracic segment, n			0.539
Yes	197(60.2)	124(62.9)	
No	130(39.8)	73(37.1)	
Lower thoracic segment, n			0.491
Yes	118(36.1)	77(39.1)	
No	209(63.9)	120(60.9)	
Ventral segment, n			0.069
Yes	1(0.3)	4(2.0)	
No	326(99.7)	193(98.0)	
Circumference, n			<0.001*
<2/3	301(92.0)	124(62.9)	
≥2/3	26(8.0)	73(37.1)	
Preoperative invasion depth, n			<0.001*
m1–m3	304(93.0)	148(75.1)	
sm	23(7.0)	49(24.9)	
Depth of pathological infiltration, n			<0.001*
m1–m3	314(96.0)	170(86.3)	
sm	13(4.0)	27(13.7)	

Circumference, total length, and preoperative invasion depth were selected to enter the multivariable analysis. Collinearity diagnostics indicated that the variance inflation factors (VIF) of predictors were all less than 2, implying no significant collinearity among them (Table 3). In the derivation set, multivariate logistic regression confirmed the three risk factors were significant predictors of ESD difficulty (Table 3). Among them, the preoperative invasion depth was the strongest risk indicator with an OR of 3.744 (95% CI, 2.077–6.747; *p* < 0.001). Compared with *a* < 2/3 circumference of the lesion, *a* ≥ 2/3 circumference of the lesion had an OR of 3.509(95% CI, 2.016–6.108; *p* < 0.001). Table 3 also revealed that there was a positive trend between the total length (OR, 1.268; 95% CI, 1.167–1.377; *p* < 0.001) and risk of technical difficulty.

**Table 3.** Collinearity diagnostics and multivariate logistic regression analysis of risk factors for ESD difficulty.

	B	OR	95% CI	*p* Value	VIF	1/VIF
Total length	0.238	1.268	1.167–1.377	<0.001	1.296	0.772
Circumference ≥ 2/3	1.255	3.509	2.016–6.108	<0.001	1.279	0.782
Preoperative invasion depth sm	1.320	3.744	2.077–6.747	<0.001	1.030	0.971

### Scoring model development and discrimination

**Figure 1.**
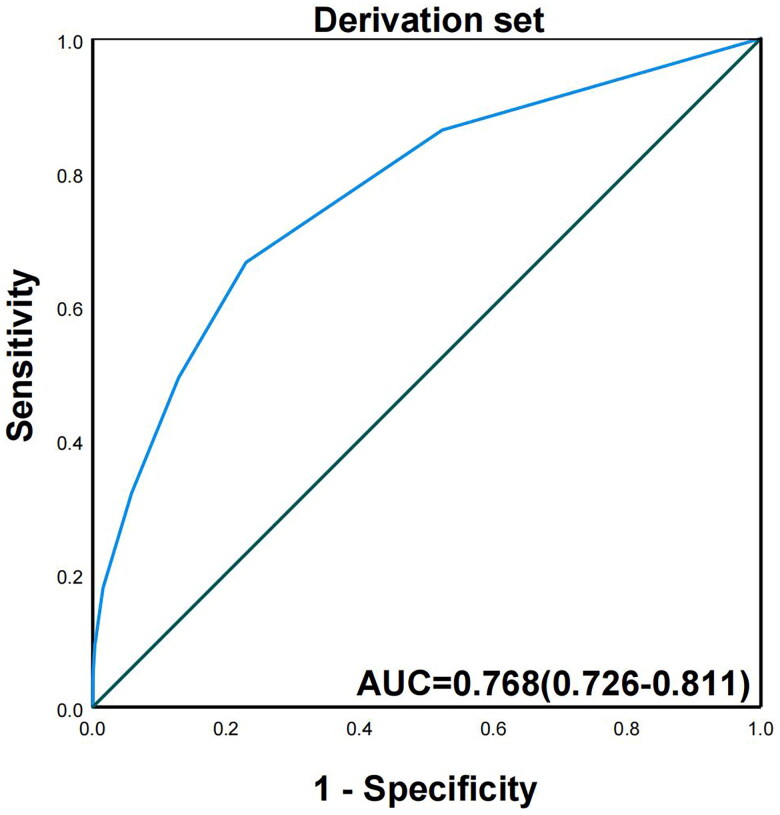
The AUC for the score model in derivation set.

**Figure 2.**
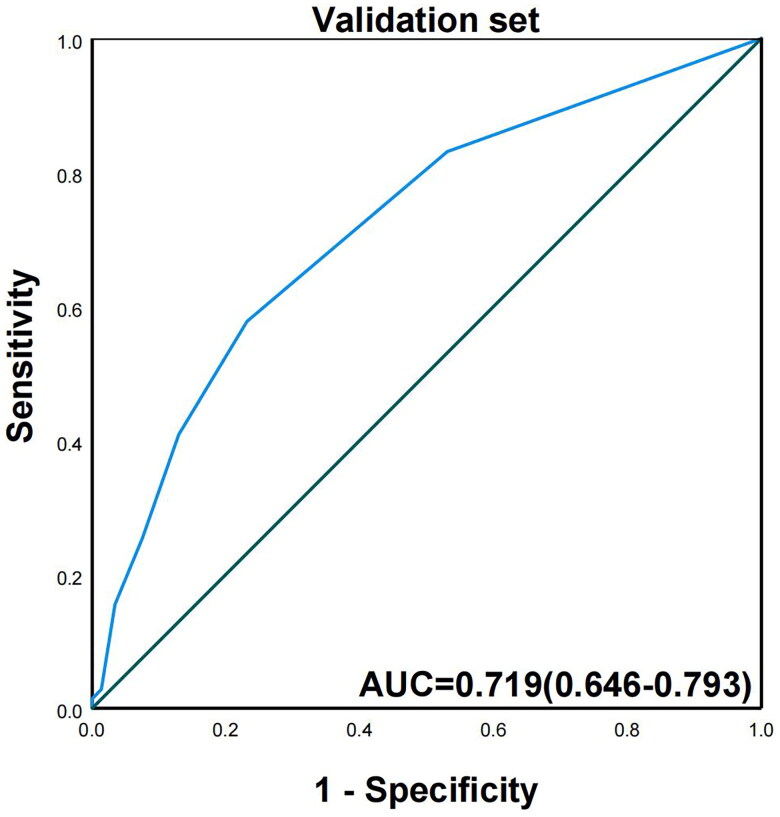
The AUC for the score model in validation set.

To establish a clinical scoring model for predicting the technical difficulty of ESD, we assigned a risk score point to each predictor based on the β (Table 4). The points of the 3 risk factors were as follows: total length 4–6 cm (1 point), 7–9 cm (2 points) or ≥10 cm (3 points); lesion ≥ 2/3 circumference (2 points); preoperative invasion depth extending to the submucosa (2 points). All applicable score values were summed to obtain a total difficulty score for each ESD operation. Based on ROC analysis, the area under the curve (AUC) for risk scores in the derivation and validation sets was 0.768 (95% CI, 0.726–0.811) (Figure 1) and 0.719 (95% CI, 0.646–0.793), respectively, indicating moderate-to-good discrimination (Figure 2).

**Table 4.** Difficulty score for independent risk factors of technically difficult ESD.

Risk factor	Categories	Reference value (Wij)	βi	βi(Wij − WiREF)	Point
Total length of lesions, cm			0.238		
	≤3	2		0	0
	4–6	5		0.714	1
	7–9	8		1.428	2
	≥10	10		2.380	3
Circumference			1.255		
	<2/3	0		0	0
	≥2/3	1		1.255	2
Tumor depth					
	m1–m3	0	1.320	0	0
	sm	1		1.320	2

**Figure 3.**
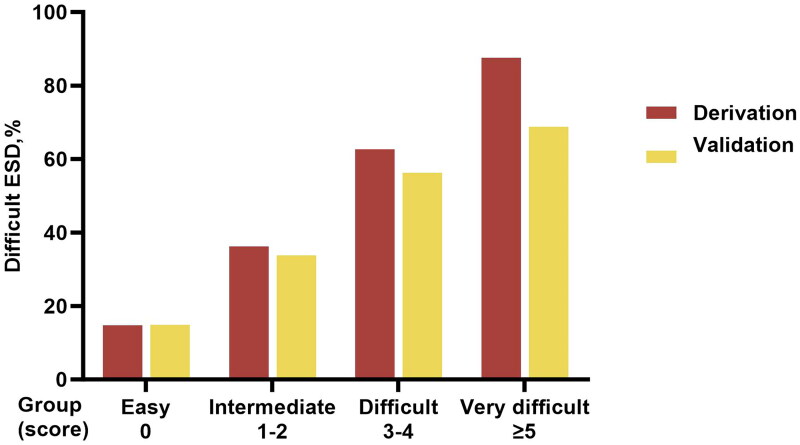
The percentage of difficult ESDs in different groups according to the prediction model.

We calculate the risk estimate for each score according to the regression equation (Table 5). The lowest total score was 0 with a risk value of 16.3% and the risk value was 96.7% for the highest score of 7. As the total points increased, the probability that the procedure would be difficult became higher. By observing the risk estimate of ESD failure within 60 min, we divided the technical difficulty of ESD into four levels: (1) easy (score = 0); (2) intermediate (score = 1 or 2); (3) difficult (score = 3 or 4); and (4) very difficult (score ≥ 5). The percentage of difficult ESDs in each group (easy, intermediate, difficult, and very difficult) is 14.8%, 36.1%, 62.6%, and 87.5% in the derivation cohort and 14.8%, 33.7%, 56.3%, and 68.8% in the internal validation cohort (Figure 3). There was a significant difference among the four groups (*p* < 0.001).

**Table 5.** The prediction probability of technically difficult ESD associated with total points.

Total point	Probability
0	0.163
1	0.285
2	0.449
3	0.624
4	0.772
5	0.874
6	0.934
7	0.967

## Discussion

Research on learning curve for ESD have shown that esophageal ESD is more technically difficult technique compared to gastric ESD, with a slower resection speed and a higher probability of complications [8, 9]. Although previous studies have reported several predictors of technically difficult ESD, including the location, tumor size, circumference, and so on, we did not find one preoperative prediction model for esophageal ESD technical difficulty [10, 11]. Therefore, the purpose of our study was to develop a risk-scoring model for grading the difficulty of esophageal ESD to help arrange surgical operators and reduce the incidence of adverse events.

ESD procedure time has been reported to be associated with failure of en bloc resection and complications, such as hemorrhage and perforation [12]. Difficult ESDs often take longer time because the endoscopists have to carefully consider each step to avoid adverse events. In addition, several studies have utilized the time parameter as the end point for defining technically difficult ESDs [13–15]. Accordingly, we chose the procedure time as one of the criteria to assess the degree of ESD difficulty. Hazama et al. reported the median procedure time in their study was 40 min which referred to the time from marking to the complete resection [11]. In comparison, the longer median procedure time of our study is reasonable because it also includes the time for the treatment of adverse events like clipping of the muscularis propria injuries and hemostasis of traumatic surfaces. In order to distinguish the really difficult ESDs, we selected 90 min, the 75th percentile of the procedure time, as the separation point. Moreover, the management of complications and incomplete resection also inevitably increases the difficulty of ESDs. Therefore, we took the intraoperative AEs and piecemeal resection into consideration when defining the difficult ESDs. In the study, en bloc resection was accomplished in all patients and no serious adverse events occurred. A total of 66 patients experienced muscularis propria injury and all received timely and successful treatment without perforation. Difficult ESDs that met the criteria accounted for 37.6% and 32.6% in the derivation and validation cohort, respectively.

The findings from this study demonstrated that lesion number, total length, the circumference of the lesion, and preoperative invasion depth were independent predictive factors affecting esophageal ESD outcomes. In terms of lesion number, it is natural that multiple lesions tend to require more time to resect. However, two small lesions are not necessarily more difficult than a single large lesion in clinical practice. The effect of the lesion number on the ESD difficulty can be reflected by the total length to some extent. Therefore, we excluded the lesion number from the prediction model.

Consistent with the results of several previous studies, the circumference of the lesion and the total length of lesions were also risk factors for the technical difficulty of esophageal ESD. For lesions with a circumference ≥ 2/3, total length ≥ 4 cm, especially ≥ 10 cm, ESD was more likely to become technically difficult. Hazama et al. found that lesions extending to more than half of the circumference were a significant factor associated with a difficult ESD [11]. Another study demonstrated ≥ 3 cm lesion length remained as factors contributing to technical difficulty performing [16]. The same conclusion was reached in the report by Hamada Y et al. [14] Larger tumor size and circumference predict more submucosal injections during the procedure and longer time for the same speed of resection. The operator needs to constantly and carefully adjust the cutting direction to ensure that the lesion is removed in one piece [13]. Noguchi et al. reported that mucosal deficiency becomes more extensive as the tumor expands, which is a strong risk factor for intraoperative perforation [17]. In addition, large tumor size has been reported to be associated with a high incidence of mucosal fibrosis [18]. An effective intraoperative fluid cushion cannot be formed due to mucosal fibrosis, which will increase the probability of hemorrhage, muscle damage, and perforation during dissection. For some lesions where mucosal fibrosis is too severe to be excised as a whole, the requirement of piecemeal resection often takes a longer time [19].

The depth of infiltration deeper than the submucosa was another independent risk factor for esophageal ESD technical difficulty in the current study. When the lesion invades the submucosa, the carcinoma cells are very close to the muscularis propria, which makes it more challenging for the operators to completely remove the lesion and ensure the integrity of the esophagus wall during surgery, thus causing damage to the esophageal tissue and increasing the risk of perforation. Since our study aimed to use preoperative information to predict ESD difficulty, in terms of the depth of tumor invasion, we referred to the results reported by preoperative EUS instead of histopathologic diagnosis. The sensitivity and specificity of EUS diagnosis in the study were 78.8% and 91.9%, respectively, which implied good diagnostic efficiency. Despite the inevitable differences between EUS and pathology, the accuracy of infiltration reported by EUS has been dramatically enhanced with the constant advance of devices and technology [20].

By quantifying these characteristics, we constructed a clinical scoring model for the difficulty of esophageal ESD. Indeed, this is the first study to develop a risk-scoring model to predict ESD technical difficulty with preoperative information. By developing this scoring model in the derivation cohort and validating it in the validation cohort, we have demonstrated the reliability and reproducibility of this system. In addition, there are unique advantages in clinical applications to this study. First, it is hoped that this model will contribute to a reasonable arrangement of ESD in a majority of centers. For hospitals with economic, technical, and equipment pressures, there is the option of transferring patients to high-volume centers that are capable of performing the difficult ESD. Second, Goda et al. recommended that esophageal ESD should be started from small (<2 cm in diameter or occupying <1/2 of the circumference) lesions for novices [21]. By classifying the level of ESD difficulty, the skills training center can assign the appropriate procedure to different experienced operators to accelerate their learning. In addition, for technically difficult esophageal ESD, having an experienced endoscopist perform the procedure can help shorten the procedure time and reduce the probability of complications, which effectively protects the benefits of the patient. Third, by predicting the difficulty and time needed for the procedure, endoscopists and anesthetists can have an initial preoperative judgment and better communicate with patients, which is helpful in increasing their trust and reducing doctor-patient disputes.

The limitations of our study are as follows: (1) Compared to a multicenter, large sample prospective study, this was a retrospective study undertaken at a single center which may affect the accuracy in predicting the difficulty of ESD. On the one hand, our center is the top-ranked hospital in our province and tends to receive patients who are difficult to handle in primary hospitals. The potential for selection bias in patient enrollment needs to be taken seriously. On the other hand, the endoscopists performing the procedure already received strict personnel training and had some experience with esophageal ESD. For the same size tumor, experienced endoscopists tend to have faster resection and lower complication rates than novices. However, the impact of the operative staff selection on the procedure duration was not included in this study. Therefore, the generalizability of our model needs to be confirmed by large amounts of different populations and endoscopists from other centers. (2) The intraoperative submucosal injection solution used in all patients in this study was a mixture of saline solution, adrenaline, and indigo carmine, whereas glycerol fructose or sodium hyaluronate are shown to be superior in maintaining mucosal augmentation over time [22]. In addition, some researchers have demonstrated that the submucosal tunneling endoscopic resection (STER) can help maintain the integrity of the mucosa and shorten surgery time [23, 24]. Another study reported the application of various traction methods during procedures is more conducive to exposing the submucosa and enlarging the surgical view, thus facilitating the operation of endoscopists [25]. However, the methods mentioned above were not used in the patients in our study. (3) The assessment of infiltration depth by EUS is subjective, which may bias the model to some extent. More relevant predictors such as preoperative biopsies should be incorporated to optimize the model in the future.

In conclusion, the clinical scoring model which incorporated three risk factors (total length, circumference, and depth of infiltration) was an available tool to evaluate the technical difficulty of esophageal ESD preoperatively. With the application of this scoring system, we can rationalize procedure planning and provide continuing education for the next generation of endoscopists according to the level of ESD difficulty.

## Data Availability

The data that support the findings of this study are available from the corresponding author upon reasonable request.
